# The Role of Vitamin D in Sleep Disorders of Children and Adolescents: A Systematic Review

**DOI:** 10.3390/ijms23031430

**Published:** 2022-01-27

**Authors:** Federica Prono, Katerina Bernardi, Raffaele Ferri, Oliviero Bruni

**Affiliations:** 1 Child Neurology and Psychiatry Unit, Department of Human Neurosciences, Sapienza University, 00185 Rome, Italy; federica.prono@uniroma1.it (F.P.); katerina.bernardi@uniroma1.it (K.B.); 2 Sleep Research Centre, Department of Neurology IC, Oasi Research Institute—IRCCS, 94108 Troina, Italy; rferri@oasi.en.it; 3 Department of Developmental and Social Psychology, Sapienza University, 00185 Rome, Italy

**Keywords:** vitamin D, sleep, insomnia, obstructive sleep apnea, restless legs syndrome, parasomnias

## Abstract

This review investigates the association between vitamin D and sleep disorders. Vitamin D is an essential nutrient known to play an important role in the growth and bone health of the human body, but it also appears to play a role in sleep. The goal of our review is to examine the association between vitamin D and sleep disorders in children and adolescents. We summarize the evidence about the role and the mechanism of action of vitamin D in children and adolescents with sleep disorders such as insomnia, obstructive sleep apnea (OSA), restless legs syndrome (RLS), and other sleep disorders. Systematic electronic database searches were conducted using Pubmed and Cochrane Library. The Preferred Reporting Items for Systematic Reviews and Meta-Analyses (PRISMA) guideline was followed. The studies that met the established inclusion criteria were analyzed and compared. Results suggest a strict relationship between vitamin D deficiency in children and sleep disorders. There is evidence that vitamin D is implicated in the different neurochemical mechanisms involved in sleep regulation and mainly in the serotonergic and dopaminergic pathways. This might be responsible for the association of vitamin D deficiency and restless sleep, sleep hyperhidrosis, OSA, and RLS.

## 1. Introduction

Vitamin D is a fat-soluble vitamin, mainly synthesized in the body through ultraviolet B (UVB) exposure on the skin or taken orally through food and/or supplements. According to the definition of the Endocrine Society we can define the following categories: deficiency (<20 ng/mL); insufficiency (between 20 and 29 ng/mL); and sufficiency (≥30 ng/mL) [1]. Vitamin D deficiency/insufficiency is a global epidemic, estimated to affect over one billion people worldwide [2], including children [3].

Even if its principal function is bone homeostasis regulation, vitamin D is also involved in several other conditions, such as cardiovascular diseases, cancer, diabetes mellitus, and autoimmune disorders [4]; recently, an increasing number of studies are showing the link between vitamin D and sleep. Low vitamin D levels have been reported to be associated with shorter sleep duration [5], and adequate levels of vitamin D seem to be necessary for the maintenance of sleep, reducing the number of nocturnal awakenings [6].

Although the exact mechanism by which vitamin D affects sleep regulation is still unclear, the key to this link seems to be the expression of vitamin D receptors (VDRs) in areas of the brainstem that are involved in sleep regulation [7,8]. Previous studies have shown that VDRs are expressed in both developing and adult rat brains [9]; in the human brain, the VDR distribution has been described as strikingly similar to that detected in rodents [10]. VDRs are expressed in the cortical and subcortical areas involved in sleep control, such as: (a) the prefrontal cortex, which mediates normal sleep physiology, dreaming, and sleep-deprivation phenomena and is activated during Non-Rapid Eye Movement (NREM) and deactivated during Rapid Eye Movement (REM) sleep [11]; (b) the cingulate gyrus, which is activated by breathing and blood pressure changes affected by sleep apnea [12]; (c) the hippocampal dentate gyrus, where neurogenesis is significant in adults [13] and is influenced by sleep deprivation, that reduces proliferation of progenitor cells [14]; (d) the caudate nucleus, which is downregulated in disturbed sleep and insomnia, especially during executive functioning [15]; (e) the lateral geniculate nucleus, which plays a major role in ponto-geniculo-occipital waves during REM sleep [16]; and (f) the substantia nigra, where the dopaminergic pathway is closely involved in the regulation of the sleep–wake cycle and is implied in idiopathic REM sleep behavior disorder [8,17].

Vitamin D might exert its effects on neurocognition based on several mechanisms mediated by sleep, including induction of neuroprotection, modulation of oxidative stress, regulation of calcium homeostasis, and suppression of inflammation [18]. Vitamin D has been proposed to act as a membrane antioxidant. In fact, it increases the gene expression levels of antioxidants agents [19] and decreases cytokine generation via inhibitory effects on the activation and expression of nuclear factor kappa B (NF-κB) and other related genes [20].

Similar to the other steroid hormones, vitamin D binds to its nuclear receptors, VDRs, and retinoid X receptors (RXRs), to effect transcriptional changes. Pertinent to sleep, VDRs and RXRs have been shown to downregulate transcription of RelB gene, a gene encoding a family of transcription factors; collectively referred to as NF-κB [21] that plays a pivotal pro-inflammatory role, both in terms of the production of sleep-regulating substances, such as IL-1 and tumor necrosis factor alpha (TNF-a), but also in terms of the selective activation of inflammatory pathways known to occur in the setting of intermittent hypoxia, as in obstructive sleep apnea (OSA) [22,23].

Since the vitamin D receptor is expressed on immune cells (B cells, T cells, and antigen presenting cells) and these immunologic cells are all capable of synthesizing the active vitamin D metabolite, vitamin D can modulate the innate and adaptive immune responses. Deficiency in vitamin D is associated with increased autoimmunity as well as an increased susceptibility to infection [24].

On the other hand, substances of the immune system, in particular the cytokines IL-1 and TNF, and prostaglandin (PG) D2 are involved in the regulation of physiological sleep in animals, and sleep duration (short and long) and disturbances (including insomnia) are linked to dysregulation of inflammatory markers, immune cell counts, and cellular aging markers. In disorders characterized by immune dysregulation, immune-therapy may not only be used to improve disease activity, but also to directly improve sleep [25,26]. Similarly, vitamin D supplementation should be considered for the prevention and treatment of immune diseases as well as for improving sleep quality.

Melatonin has also been suggested to act as a mediator of the neuro-immunomodulatory properties of vitamin D [27]. Alongside Vitamin D, melatonin participates in the regulation of circadian rhythms and sleep, immune response, and bone metabolism [28,29]. Melatonin and its metabolites exhibit a wide spectrum of both direct and indirect physiological effects in humans [30,31,32,33,34]. First, these compounds scavenge free radicals and other non-radical Reactive Oxygen Species/Reactive Nitrogen Species (ROS/RNS) directly, reducing the level of oxidative stress and, thus, show antioxidant abilities preventing inflammation. Second, these biomolecules participate in immunomodulation, improve immune defense, and exhibit other physiological activities, e.g., regulate circadian rhythms, body temperature, increase physical performance and glucose uptake in muscles, and prevent against lipid accumulation, among others [35,36,37,38,39]. Importantly, melatonin is effective in adjusting the sleep–wake cycle and improving the quality of sleep. Melatonin stabilizes circadian rhythms and exerts its chronobiotic effects by acting on the plasma membrane trough G protein-dependent receptors type 1 and type 2 called MT1 and MT2, and its rhythmic release is regulated by a central circadian rhythm generator [40].

### 1.1. Vitamin D and the Serotonergic System

Soon after the time of its discovery, over 40 years ago, the serotonergic system was implicated in the regulation of the sleep–wake cycle. While early studies indicated that serotonin (5-HT) was associated with the initiation and maintenance of sleep, later studies indicated that serotonergic neurons also play a role in inhibiting sleep. The complex effects of 5-HT on the regulation of sleep are due in part to the fact that 5-HT can act at different areas of the brain that have been associated with the control of sleep and wake.

In addition, the recent discovery of multiple 5-HT receptors within the mammalian brain has led to the finding that different 5-HT receptors are selectively involved in the regulation of the different sleep states [41,42]. Based on electrophysiological, neurochemical, genetic, and neuropharmacological approaches, it is currently accepted that 5-HT functions predominantly to promote wakefulness and to inhibit REM sleep. However, under certain circumstances this neurotransmitter contributes to the increase in sleep propensity [43]. Serotonin is synthetized in the brain from its precursor tryptophan, an essential amino acid obtained from the diet [44,45] through the action of tryptophan hydroxylase and participates in the regulation of circadian rhythms [46,47,48].

Vitamin D plays a key function in the regulation of the serotonergic pathway [18,49,50] and in melatonin production, confirming the importance of vitamin D in sleep but also in mood regulation [50,51]. Furthermore, the presence of VDRs in limbic structures, including hippocampus, amygdala, and prefrontal cortex, suggests that vitamin D could be also associated with the regulation of mood and emotional behavior [52]. In detail, vitamin D can influence the serotoninergic pathway in the brain and in the peripheral tissues binding the vitamin D response elements (VDREs) on the tryptophan hydroxylase genes (THP1 and THP2), involved in serotonin production. Vitamin D inhibits the expression of THP1 in the peripheral tissues and increases the expression of THP2 in the brain [50,53]. A special isoform of the enzyme tryptophan hydroxylase, TPH2, converts the amino acid tryptophan into 5-hydroxytryptophan, a precursor of serotonin. TPH2 is entirely restricted to neurons of the raphe nuclei and the enteric nervous system and is the enzyme responsible for producing all of the serotonin in the brain [54].

Serotonin in the brain promotes prosocial behavior and correct assessment of emotional social cues [55]. This seems to explain the link between vitamin D levels, serotonin, sleep, and mood regulation [53]. Regarding sleep, vitamin D exerts an important function acting on the THP1 expression in the pineal gland. Through THP1 expression, the pineal gland converts serotonin into melatonin during evening and nighttime [56,57]. According to the daily variation in natural light exposure [58], the variation of serum vitamin D levels, from relatively high during daytime to relatively lower during nighttime, may be necessary for optimal TPH1 expression in the pineal gland for melatonin regulation. It may be the case that disturbances in these daily variations could have an influence on sleep timing and/or quality [51]. In addition, vitamin D regulates the conversion of tryptophan into 5-HTP [50] and regulates the production of melatonin also for its action on tryptophan hydroxylase [59].

### 1.2. Vitamin D and the Dopaminergic System

On the other hand, vitamin D plays an important function in the dopaminergic system participating in the regulation of the nervous system development and function [60]. Dopamine neurons in the midbrain and their target neurons in the striatum were shown to express vitamin D receptor proteins, and vitamin D receptors are present in the nucleus of tyrosine hydroxylase-positive neurons, in both, human and rat substantia nigra [61]. Oran et al. [62], observed how rat primary dopaminergic neurons had a dose-responsive increase in number when vitamin D3 (the hormonally active form of vitamin D) was added to culture media, suggesting that vitamin D might increase the number of dopaminergic neurons by upregulating the expression of glial-derived neurotrophic factor. In addition, it has been reported that vitamin D affects the nigrostriatal dopaminergic pathway by increasing the levels of dopamine or its metabolites and by protecting dopaminergic neurons against toxins [63,64].

Treatment with vitamin D could increase dopamine concentration and its metabolites in the substantia nigra and protect mesencephalic dopaminergic neurons against toxins that cause a decrease in the glutathione content, which might lead to selective dopaminergic neuron death [65,66]. In fact, it seems that vitamin D may participate in the antioxidant mechanism controlling brain homeostasis [67]. Vitamin D has been recently reported to enhance the intracellular glutathione concentration in the central nervous system [68].

Exposing rat cultured mesencephalic neurons for 24 h to a mixture of L-buthionine sulfoximine (BSO) and 1-methyl-4-phenylpyridium ions (MPP) resulted in a relatively selective damage to dopaminergic neurons [69]. This damage was accompanied by a reduction in intracellular glutathione levels. Low doses of Vitamin D3 protect cultured dopaminergic neurons against this toxicity. Generation of ROS by this toxicity has been attenuated in cultures being pretreated with low concentrations of Vitamin D3. These data suggest that low doses of Vitamin D3 are able to protect mesencephalic dopaminergic neurons against BSO/MPP induced toxicity that causes a depletion in glutathione content [63].

It is interesting to notice that dopaminergic dysfunction, together with iron dysregulation are the main pathophysiologic mechanisms involved in the development of Restless Legs Syndrome (RLS) [70]. Vitamin D may be involved in the pathogenesis of RLS because of its effects on the dopaminergic system, through VDRs present in the nucleus of neurons that are positive for tyrosine-hydroxylase in the substantia nigra. In fact, Vitamin D deficiency can be considered a risk factor for RLS. The incidence of RLS, indeed, is increased in adult patients with vitamin D deficiency [61,70,71] and a significant inverse correlation was found between vitamin D levels and severity of RLS [72]. Interestingly, infants diagnosed with iron-deficiency anemia simultaneously show low levels of serum vitamin D [73]. The addition of vitamin D to their diet might improve blood and tissue iron concentration.

Therefore, due to his effect at the gene and receptor levels, it is not surprising that vitamin D might exert a clinical effect on sleep and sleep disorders. The impact of vitamin D on sleep has been well described in adults; its deficiency has been associated with multiple sleep disorders such as OSA, RLS, changes in sleep duration, and worsening of sleep quality [22]. Adult patients return to normal sleep cycles with vitamin D levels at 60–80 ng/mL suggesting the need to reach levels higher than the normal accepted values of 30 ng/mL for the treatment of sleep disorders.

Regarding the pediatric population, there are only few studies on the correlation of vitamin D deficiency and sleep disorders. Due to the beneficial effect of vitamin D supplementation in adults, it might be expected that vitamin D supplementation might also improve sleep in children and adolescents with sleep disorders. In this review we aimed to outline the experimental evidence of the role of vitamin D in the regulation of sleep, mainly duration and quality of sleep and in sleep disorders, such as OSA, RLS, and insomnia in children and adolescents [74,75].

## 2. Materials and Methods

### 2.1. Search Strategies and Selection of the Studies

**Figure 1:**
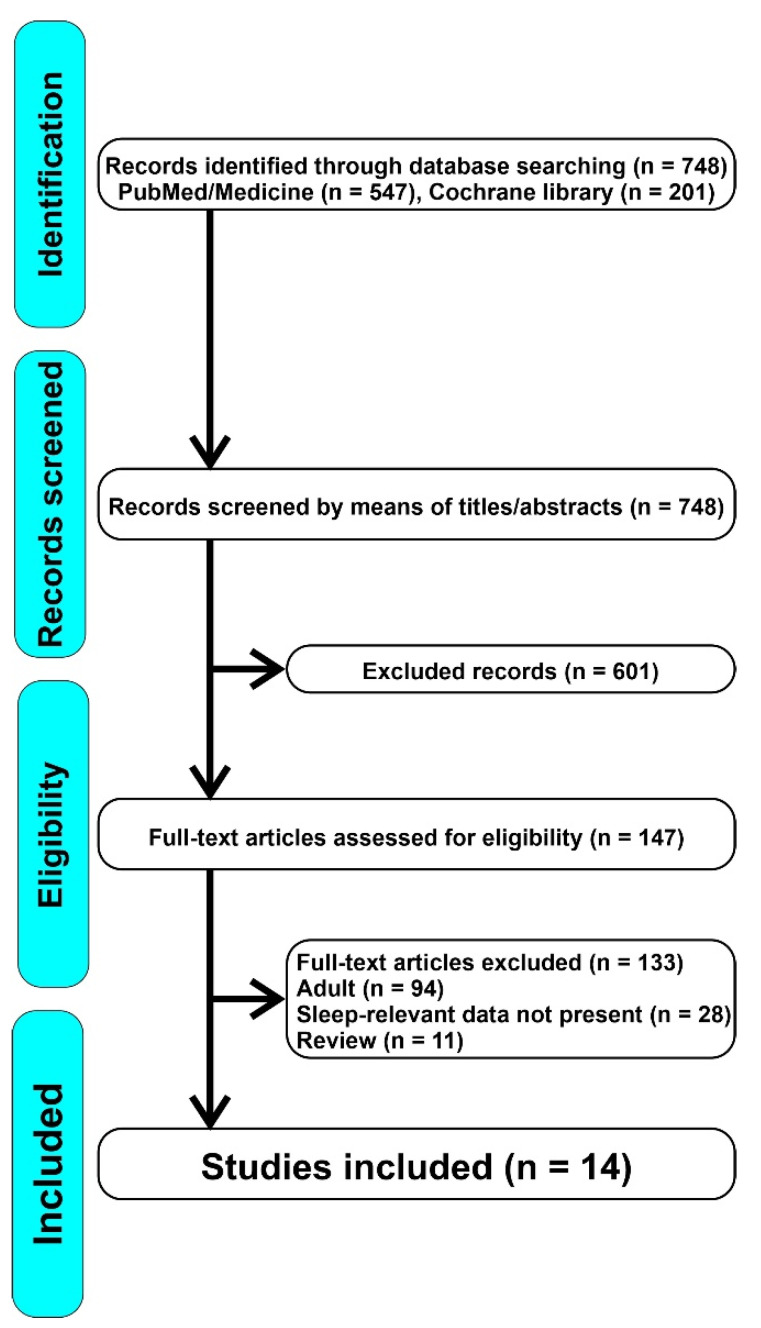
Overall flowchart of the articles screened.

The review was conducted according to the PRISMA guidelines. Two electronic databases (PubMed and Cochrane Library) were systematically analyzed. For both databases, search terms included the following combination of keywords: “vitamin and sleep OR vitamin D and insomnia OR vitamin D and OSA OR vitamin D and RLS OR vitamin D and parasomnias”. Retrospective-cohort, cohort, prospective, observational cross-sectional, case-control, prospective and comparative, multi-center cross-sectional, and longitudinal studies were included in the present systematic review. No restrictions were applied to the publication period or to the country in which the study was conducted. No filters were used to avoid the loss of potentially interesting documents. Following the PRISMA method, we screened the articles by means of keywords, titles, and abstracts. Before proceeding, duplicate documents were filtered out. After the first screening, we excluded irrelevant articles; subsequently, we carried out an analysis of the full text articles to select the most appropriate ones. In the first instance, we also evaluated articles that would give us a more complete picture of the mechanisms of action and the relationship between vitamin D and sleep, even in the adult population, which has been studied more often. We afterwards excluded reviews, articles that dealt with the adult population or in which sleep data were not relevant to our research or articles in which a close correlation between sleep and vitamin D was not explicitly tested. The study selection flowchart is illustrated in Figure 1.

### 2.2. Data Extraction and Quality Assessment

Three authors (O.B., F.P. and K.B.) independently assessed the articles and extracted the data and disagreements were resolved through discussion. Extracted Information included: (1) title; (2) the last name of the first author; (3) publication year; (4) objective; (5) study design details; (6) study population characteristics; (7) methods; and (8) results. The content and methodology of the studies were analyzed qualitatively, summarizing the main findings according to the study purpose. To ensure reliability, articles selected by the first author were assessed by a second independent researcher. Furthermore, the level of evidence was assessed. Papers that seemed to meet the inclusion criteria but caused doubt due to ambiguities were analyzed once more by a third investigator until consensus was reached. In view of the scarcity of papers in the available literature, none of the studies was excluded due to quality issues.

## 3. Results and Discussion

We identified 748 articles, of which 601 were excluded because they were not relevant for our research; therefore, we assessed 147 full-text articles for eligibility, from which we excluded 133 because they focused on the adult population or because the sleep data were not pertinent. Ultimately, we included 14 articles in our review. Table 1 shows a summary of the main data reported by the studies included: type of study, objective, sample, methods, and results. For the purpose of this review, we divided the included articles in three main groups based on the sleep disorder considered (sleep duration and quality of sleep, OSA, and other sleep disorders).

### 3.1. Vitamin D and Sleep Duration and Quality of Sleep

#### 3.1.1. Association between Sleep Duration and Plasma Vitamin D Levels in Children

Gong et al. [76], examined the association between 25-Hydroxyvitamin D (25(OH)D) levels and sleep duration among 800 Chinese adolescents aged 8–14 years. They analyzed anthropometric measurements by trained research staff, serum vitamin D and lipids were measured in the laboratory, and sleep habits and other health-related behaviors were tested by questionnaires. Nearly one-third (32.8%) of the subjects were sleep insufficient (sleep duration < 9 h per day), and 30.3% were vitamin D insufficient (serum level < 20 ng/mL). There was a small correlation between sleep duration and the concentration of vitamin D in this study (r = 0.11, *p* < 0.05). The authors finally suggested that vitamin D status could be a potential biomarker of insomnia or lack of sleep in children.

Al-Shawwa et al. [75] examined the relationship between sleep architecture and vitamin D status in children. They conducted a retrospective-cohort study with 39 patients aged 2–17 years (mean age 6.6 years; 46% female) in a tertiary care children’s hospital over a 1-year period. They included children who underwent an in-laboratory overnight polysomnogram and had a 25(OH)D level obtained within 120 days from the sleep study. Patients with OSA or central sleep apnea were excluded. Twenty children (51%) had vitamin D deficiency (25(OH)D level < 30 ng/mL) and had less total sleep time (470.3 ± 35.6 min vs. 420.3 ± 61.7 min; *p* = 0.004) and poorer sleep efficiency (91.9 ± 5.6% vs. 84.5 ± 9.5%; *p* = 0.015) compared to children with sufficient vitamin D. In addition, children with vitamin D deficiency had later weekday bedtimes (21:02 ± 1:01 h vs. 20:19 ± 0:55 h; *p* = 0.037) and later weekend bedtimes (21:42 ± 0:59 h vs. 20:47 ± 1:08 h; *p* = 0.016). This study suggests that vitamin D deficiency in children is associated with objectively measured decreased sleep duration and poorer sleep efficiency. Furthermore, vitamin D deficiency was associated with delayed bedtimes, suggesting that vitamin D and circadian rhythm could be related.

Sung et al. [77], examined 618 10- to 12-year-old children, 111 (18.0%) with excessive daytime sleepiness (EDS), and 507 (82.0%) healthy controls. The two groups had no significant differences in age, sex, body mass index (BMI) z-score, weight status, birth weight, and presence of allergic diseases. Children with low vitamin D levels (<20 ng/mL) had an increased risk of EDS (adjusted OR = 1.73; 95% CI 1.06–2.81; *p* = 0.028). Low vitamin D level, lack of exercise, and high BMI were the most important factors contributing to EDS, of which low vitamin D level was the strongest one. Additionally, vitamin D deficiency had a strong relationship with shorter sleep duration and less sleep efficiency after adjusting for BMI and age, but no relationship with sleep stages, periodic limb movements, and arousal index at polysomnography [75]. The authors finally suggested that vitamin D level might play a crucial role in predicting the severity of EDS, and vitamin D supplements could be used, as an example, to treat school children with EDS.

Valtuerna et al. [78] and Zhao et al. [79], evaluated the factors associated with vitamin D deficiency in adolescents and preschool children and found out that sleep duration was one of the most relevant factors associated with vitamin D serum levels. Valtuerna et al. [78] examined 1006 European adolescents (aged 12.5–17.5 years) in a multicenter study measuring body composition, biochemical marker, socioeconomical status, dietary intake, physical activity, fitness, sleep time, and vitamin D genetic polymorphism in a stepwise multivariate linear regression analysis stratified by gender. The results showed that sleep duration was one of the factors that strongly influenced vitamin D concentrations in adolescents (together with latitude, season, adiposity, fitness, and micronutrient supplementation).

In the Jiangsu Bone Health Study, Zhao et al. [79] assessed the vitamin D status with its demographic and lifestyle factors in 5289 children during the first 5 years of life in a population-based cross-sectional multicenter study in China. The prevalence of vitamin D deficiency was 30.1%. Children with sleep duration < 10 h had higher odds of vitamin D deficiency and a lower 25(OH)D concentration (all *p* < 0.05).

In summary, vitamin D deficiency has been shown to be associated with decreased sleep duration and poorer sleep efficiency, as well as with delayed bedtimes [75]. Moreover, children with reduced vitamin D serum levels have a higher risk of EDS when compared with the general population [77]. Since vitamin D levels influence sleep duration, sleep duration can also influence vitamin D serum concentration [78], suggesting a bidirectional relationship between vitamin D and sleep duration. Although this relationship seems to be quite strong, other factors might influence sleep duration and quality, so that vitamin D deficiency not alone, but in coaction with other factors, might cause sleep disorders.

#### 3.1.2. Correlation between Cord Blood Vit D Levels and Sleep Features of Preschool Children

Yong et al. [80], explored the association between cord-blood vitamin D level at birth and night-sleep duration trajectories in children aged between 2 and 5–6 years, in a non-clinical cohort. They analyzed 264 children presenting available data for both vitamin D measures determined at birth and sleep trajectory (using parental self-administered questionnaires). They showed that the vitamin D pool of the fetus and newborn depends on their mother vitamin D status; for this reason, hypothetically, vitamin D supplementation during pregnancy might reduce vitamin D deficiency in infants and might favor both brain development and healthy sleep in children. This article also suggests that a low vitamin D level at birth is associated with increased odds of children aged between 2 and 5–6 years to be persistent short sleepers.

Deng et al. [81], explored the association between vitamin D in cord blood or in venous blood and children’s sleep–wake patterns at two years of age. Data were obtained from 209 children in a birth cohort, Shanghai Sleep Birth Cohort Study. Vitamin D was assessed in cord blood and venous blood samples by electrochemiluminescence immunoassay. Children’s sleep–wake patterns were measured with the Brief Infant Sleep Questionnaire and objectively with actigraphy. The prevalence of vitamin D deficiency (defined as <50 nmol/L) was 50.4% in cord blood and 28% in venous blood. This suggested that the cord blood vitamin D level was not significantly associated with children’s sleep at two years of age. On the other hand, children with vitamin D deficiency had shorter reported and actigraphic night sleep duration and total sleep duration than those with normal vitamin D concentration, so vitamin D level was positively associated with night and total sleep duration.

In conclusion, not the cord blood but rather the venous blood vitamin D level was associated with children’s sleep–wake patterns, at two years of age. To summarize, from these studies we can deduce that mothers’ vitamin D levels during pregnancy are important for the determination of the vitamin D pool of the fetus and of the newborn. Indeed, low vitamin D levels at birth expose children between 2 and 6 years to an increased risk to be persistent short sleepers [80]. Nevertheless, Deng et al. [81] showed that not the cord blood vitamin D level but rather the venous blood vitamin D level was associated with children’s sleep–wake patterns, at two years of age.

### 3.2. Vitamin D and OSA

OSA in children is a disease characterized by recurrent episodes of partial or complete upper airway obstruction associated with arousals, awakenings, and/or oxyhemoglobin desaturations during sleep. It may also be associated with disruption of ventilation and normal sleep patterns [82]. If inadequately diagnosed/treated in children, it can be associated with behavioral problems, learning difficulties, cardiovascular complications, and growth retardation [83,84]. OSA is a relatively common disorder in childhood affecting up to 3% to 4% of all children. Two studies (Kheirandish-Gozal et al. [74] and Ozgurhan et al. [85]) demonstrated a linear relationship between vitamin D levels and risk of OSA. In their study, Kheirandish-Gozal et al. [74] hypothesized that OSA might be associated with lower vitamin D levels and increased risk of metabolic dysfunction and systemic inflammation.

The role of vitamin D in systemic inflammation has been investigated in adults. Lower vitamin D serum levels have been associated with an increased risk of respiratory infection and an increased incidence of allergic rhinitis. Recurrent respiratory infections and immune system dysregulation may promote the development of tonsillar hypertrophy and chronic rhinitis, both of which increase the risk of OSA. Furthermore, OSA has been described as a low inflammatory state disease and vitamin D might be helpful by inhibiting the secretion of proinflammatory T-helper cell 1 cytokines IL-2, IFN-g, and TNF-a and enhancing the production of anti-inflammatory Th2 cytokines (IL-3, IL-4, IL-5, and IL-10) [86].

In another study, Ozgurhan et al. [85], evaluated the risk of OSA in two groups of children according to their levels of vitamin D: a low-level vitamin D group (<20 ng/mL) and a control group (>20 ng/mL). The risk of developing OSA as determined by the Berlin Questionnaire was found to be statistically higher in the low-level vitamin D group when compared with the control group (*p* = 0.030). The percentage of patients at high risk of developing OSA was 14.16% for the low-level vitamin D group and 5.83% for the control group.

Another interesting study by Zicari et al. [87] assessed the association between mean platelet volume (MPV), vitamin D, and C Reactive Protein (CRP) in patients with OSA, primary snoring (PS), and a control group. MPV levels were higher in subjects with OSA and PS when compared to controls; platelet count (PLT) and CRP levels were also higher while vitamin D levels were lower in children with OSA and PS when compared to the control group.

Other studies hypothesized that vitamin D might play a role in modulating behavioral and cognitive dysfunctions in children with OSA [88,89]. Cui et al. [90] found that triglycerides, total cholesterol, low-density lipoprotein, and body mass index of the OSA group were clearly higher than those of the control group, while the level of serum vitamin D and high-density lipoprotein was clearly lower. The supplementation of vitamin D determined an improvement of the vitamin D level and a decrease in the indexes of conduct problems, learning problems, and hyperactivity. Vitamin D supplementation had no therapeutic effect on obesity and dyslipidemia of OSA children but had obvious protective and improving effects on neuron damage caused by hypoxia [91].

Another interesting fact that emerges from our review is that the level of vitamin D in parents can play a role in determining the blood levels of vitamin D in children with snoring problems. This correlation was analyzed by Barceló et al. [92] who assessed the interrelationship between serum vitamin D levels and metabolic profiles, sleep parameters and paternal and maternal vitamin D status in a sample of snoring children referred to a sleep unit. Significant associations were found between serum vitamin D concentrations in children who snored and their parents. The prevalence of vitamin D insufficiency of the parents varied significantly based on the children’s vitamin D status and was greater in parents whose children had vitamin D insufficiency: overall in 64.9% of fathers and 63.2% of mothers. In children with vitamin D deficiency, an inverse correlation between the apnea–hypopnea index and respiratory arousal index and vitamin D concentrations was also observed. This study suggests that a familial status of vitamin D could be used as an indicator for the early identification of children at risk of unhealthy sleep and/or metabolic complications.

### 3.3. Other Studies on Specific Diseases

Two studies analyzed vitamin D levels in specific pediatric groups: with celiac disease (CD) and with Familial Mediterranean Fever (FMF). In the first study, the goal was to determine the prevalence of RLS in children with CD and to investigate the associated factors for RLS, such as iron and vitamin D levels. CD is an immune-mediated enteropathy triggered by ingestion of dietary gluten in genetically predisposed individuals [93]. Işıkay et al. [94] studied 226 children with CD and 268 control children showing that RLS prevalence was similar in both groups (3.5% vs. 3.0%, respectively). In children with CD, RLS severity was negatively correlated with serum ferritin, folic acid, or 25(OH)D levels. The CD group with RLS showed also iron deficiency anemia.

The second study investigated the correlation between serum levels of vitamin B12 and vitamin D with the self-reported quality of sleep of pediatric patients with FMF. FMF is the most common autoinflammatory disorder, inherited in an autosomal recessive manner and characterized by recurrent fever and serositis. Ekinci et al. [95] selected 63 children with FMF divided into subgroups depending on vitamin D serum concentrations: ≥20 and <20 ng/mL and vitamin B12 serum concentrations: ≥200 and <200 pg/mL. Information on sleep quality were obtained using self-administered Pittsburg Sleep Quality Index (PSQI) questionnaire. Total PSQI score, sleep disorders, and daytime sleepiness sub-scores were statistically higher in patients with serum vitamin D levels below 20 ng/mL. Vitamin D deficiency was present in 36.5% of patients and low levels of vitamin D correlated with poorer sleep quality.

## 4. Conclusions

The present systematic review, to our knowledge, is the first to assess the association between Vitamin D and sleep disorders in children. However, some potential limitations should be recognized: (a) the number of studies eligible for our review was small and with different study designs; (b) most of the studies were cross-sectional; and (c) there was a high heterogeneity of the studies linked to different assessment of sleep and of vitamin D deficiency. Nevertheless, this review demonstrates that Vitamin D has both a direct and an indirect role in the regulation of sleep and that vitamin D deficiency < 20 ng/mL is associated with a higher risk of sleep disorders in children. However, although vitamin D deficiency has been associated with sleep disorders, evidence is still scarce to concretely support the role of vitamin D supplementation in the prevention or treatment of sleep disorders in children. Therefore, high-quality prospective cohort studies and well-designed randomized controlled trials (RCTs) are needed to verify this relationship and to determine the effect of vitamin D supplementation in children with sleep disorders.

## Figures and Tables

**Table 1:** Summary of the main data reported by the studies considered in this review.

Study	Objective	Design	Population	Methods	Results
Al-Shawwa et al. 2020	Relationship between sleep architecture and vitD status.	Retrospective cohort study.	39 children	PSG and pediatric sleep questionnaires.	51 with vitD deficiency (25(OH)D < 30 ng/mL). Children with vitD deficiency: decreased TST and sleep efficiency, and later weekday and weekend bedtimes.
Deng et al., 2020	Association between vit D in cord or venous blood and sleep–wake patterns at two years of age.	Prospective cohort study.	29 children	25(OH)D assessed in cord blood and venous blood at two years of age. Sleep–wake patterns measured with BISQ and Acti-Watch.	Venous but not cord blood 25(OH)D level at two years age positively associated with sleep duration.
Gong et al., 2018	Association between 25(OH)D levels and sleep duration.	School-based prospective study.	800 Chinese adolescents (8–14 years)	Anthropometric measured by trained research staff. Serum 25(OH)D and lipids measured in the laboratory. Sleep habits and health-related behaviors assessed by questionnaire.	25(OH)D levels positively correlated with sleep duration. Insufficiency/deficiency of vitD (25(OH)D < 20 ng/mL) significantly associated with increased probability of short sleep.
Yong et al., 2019	Association between cord-blood vitD levels at birth and night-sleep duration trajectories between 2 and 5–6 years old.	Cohort study.	264 children	Cord-blood 25OHD determined by radio-immunoassay at birth, and night-sleep trajectories between 2 and 5–6 years obtained by group-based trajectory modeling method. Associations assessed by multinomial logistic regression adjusted for maternal and child characteristics.	Trajectories short sleep (<10.5 h) was found in 5%, medium–low sleep (10.5–11.0 h) in 46%, medium–high sleep (≈11.5 h) in 37%, long sleep (≥11.5 h) in 4% and changing sleep (decreased from ≥11.5 to 10.5–11.0 h) in 8%, respectively. The mean 25OHD level was 19, 12, 19, 14, and 16, respectively. On adjusted analysis, decrease in 25OHD level correlated with the odds of belonging to the shorter sleep trajectories.
Kheirandish-Gozal et al., 2014	Association between OSA and plasma 25(OH)D and risk of metabolic dysfunction and systemic inflammation.	Observational cross-sectional study.	176 obese and non-obese children with and without OSA	PSG and fasting blood draw the morning after. Lipid profile, homeostatic model of insulin resistance and high-sensitivity C-reactive protein assays and plasma 25(OH)D assessed.	25(OH)D levels reduced in pediatric OSA (also in Afro American and in obese children); possible role in modulating the degree of insulin resistance and systemic inflammation.
Shin et al., 2018	Relationship between vitD and associated factors in children ATH.	Retrospective cross-sectional study.	88 children with sleep-disordered breathing	Four groups based on adenoidal and/or tonsillar hypertrophy. Demographic data, the sizes of tonsils and adenoids, serum 25(OH)D level, BMI, and allergen sensitization patterns.	Children with ATH had decreased 25(OH)D). Children with vitD deficiencies higher frequency of ATH. Inverse correlation between serum 25(OH)D levels and age, tonsil and adenoid size, and height. Tonsil and adenoid size, and BMI-z score associated with 25(OH)D levels, after controlling for age, sex, height, and mite sensitization.
Ekinci et al., 2017	Serum vitB12 and vitD correlation with self-reported sleep quality of pediatric FMF patients.	Case-control study.	63 children with FMF	Self-administered PSQI. The patients divided into subgroups depending on vitD concentrations (≥20 and <20 ng/mL) or to vitB12 concentration (≥200, <200 pg/mL).	vitB12 levels not correlated with PSQI scores. Significant correlation between vitD and total PSQI scores and daytime sleepiness. Total PSQI score, sleep disorders and daytime sleepiness sub-scores higher in patients with vitD < 20 ng/mL. vitD possibly protective against sleep disorders and poor sleep.
Zhao et al., 2021	vitD status by demographic and lifestyle factors including dietary supplementation and physical activity.	Population based, cross-sectional, multicenter study.	5289 children aged 0–5 years	Stratified cluster random-sampling method in 12 Children’s Health Care Centers from 10 cities in Jiangsu Province, China.	Prevalence of vitD deficiency 30.1%. Higher risk of vitD deficiency associated with: older age, girls, survey conducted in spring, location in southern Jiangsu province, residence in urban, outdoor activity < 2 h/day. Lower risk associated with: parity ≥ 2 times, vitD supplementation from birth to 6 months, vitD supplementation starting ≤ 1 month after birth, vitD and calcium supplementation in the last 3 months, and dose of vitD supplementation > 400 IU/day. Higher risk of vitD deficiency with preference for sweets, meat consumption > 150.0 g/day1, milk consumption < 250 mL/day, sleeping < 10 h/day.
Ozgurhan et al., 2016	Risk of OSA in subjects with vitD deficiency.	Prospective and comparative study.	176 obese and non-obese children with and without OSA	Two groups based on 25(OH)D levels: low level (<20 ng/mL) group (n = 120) and control (>20 ng/mL) group (n = 120). Risk of developing OSA assessed by Berlin Questionnaire.	No statistically significant differences between the low level and control groups in terms of gender, age, and BMI z-score distributions. 24 subjects with high risk of developing OSA (17 subjects in the low-level group and 7 subjects in the control group). Risk of developing OSA significantly higher in the low-level group. BMI z-score significantly higher in high-risk groups than low-risk groups.
Cui et al., 2021	vitD in the treatment of children with OSA.	Case-control study.	50 children: 30 with OSA, 20 controls	In all subjects: sex, age, triglyceride, total cholesterol, HDL, LDL, serum 25-OHD levels, and Conners’ parental scale were measured. In children with OSA: BMI, AHI, and minimum oxygen saturation. Children with OSA treated with Rocaltrol (0.25 g/QD) for 4 weeks and reanalyzing their triglycerides, total cholesterol, HDL, LDL, serum 25(OH)D levels, sleep AHI, minimum oxygen saturation, and Conners’ parental scale.	Children with OSA frequently obese, with dyslipidemia, and vitD deficiency, with behavioral and cognitive dysfunction. No significant changes in BMI, triglycerides, total cholesterol, HDL, LDL, sleep AHI, and minimum oxygen saturation after vitD treatment, but the serum 25-OHD level significantly improved, as well as conduct problems, learning problems, and hyperactivity index decreased.
Sung et al., 2020	Factors associated with EDS and vitD level.	Case-control study.	618 children: 111 with EDS and 507 healthy controls	Physical examination, acoustic rhinometry, and blood sampling. Parent-filled questionnaires. Korean version of Pediatric Daytime Sleepiness Scale (PDSS).	Children with low 25(OH)D3 (<20 ng/mL) and HDL-C (<40 mg/dL) levels with increased risk of EDS. 25(OH)D3 level, exercise, and BMI were over three. High levels of 25(OH)D3 and HDL cholesterol and performing regular exercise associated with decreased risk of EDS.
Valtuena et al., 2013	Environmental, individual, and genetic factors associated with 25(OH)D levels.	Multi-center cross-sectional study.	1006 children	Measures of body composition, biochemical markers, socioeconomic status, dietary intake, physical activity, fitness, sleep time, and vitamin D genetic polymorphism (rs1544410).	In males, 25(OH)D levels independently influenced by winter season, higher latitudes, BMI z-score and retinol concentration. In females, 25(OH)D levels independently influenced by winter season, sleep time, supplement intake, flexibility, body fat %, BMI z-score, higher latitudes, and handgrip strength. Season, latitude, fitness, adiposity, sleep time, and micronutrient supplementation were highly related to 25(OH)D concentrations.
Işıkay et al., 2018	Prevalence and associated factors of RLS in children with CD.	Cross-sectional study: case-control study.	494 children: 226 with CD and 268 controls	Demographic data, educational status and routine laboratory data of children including complete blood count, ferritin, vitB12, folate and 25(OH)D levels. RLS prevalence and associated symptoms by a 30-item questionnaire.	Prevalence of RLS not increased in children with CD. Age at onset of RLS symptoms significantly younger and more severe in CD.
Barceló et al., 2021	Inter-relationship between serum 25(OH)D levels and metabolic profiles, sleep parameters, and paternal and maternal vitD status.	Familial longitudinal study.	137 Caucasian families (children and their parents)	Measurement of serum 25(OH)D levels, serum glucose, lipids, liver enzymes, parathyroid hormone, insulin, and glycated hemoglobin and evaluation of overnight PSG.	VitD insufficiency (<30 ng/mL) and deficiency (<20 ng/mL) in 40.9% and 17.5%, respectively. Risk of vitD insufficiency increased by both paternal and maternal insufficiency. Serum 25(OH)D concentration associated with AHI and respiratory arousal index.

Legenda: PSG = polysomnogram; vitD = vitamin D; 25(OH)D = 25-hydroxy vitamin D; TST = total sleep time; BISQ = Brief Infant Sleep Questionnaire; OSA = obstructive sleep apnea; ATH = adenotonsillar hypertrophy; BMI = body mass index,; vitB12 = vitamin B12; FMF = familial Mediterranean fever; PSQI = Pittsburg Sleep Quality Index; AHI = apnea/hypopnea index; EDS = excessive daytime sleepiness; HDL = and high-density lipoprotein; LDL = low-density lipoprotein; RLS = restless legs syndrome; CD = celiac disease.
